# Chlorido[4-chloro-2-(pyridin-2-yl­methyl­imino­meth­yl)phenolato-κ^3^ *N*,*N*′,*O*]copper(II)

**DOI:** 10.1107/S1600536812013359

**Published:** 2012-04-04

**Authors:** Haixia Wang, Yuehe Lang, Shaohong Wang

**Affiliations:** a Department of Chemistry and Environmental Science, Henan Normal University, Xinxiang 453007, People’s Republic of China

## Abstract

In the title complex, [Cu(C_13_H_10_ClN_2_O)Cl], the Cu^II^ ion is coordinated by one O atom and two N atoms of the tridentate Schiff base ligand and one chloride ion, forming a slightly distorted square-planar geometry. Weak Cu⋯Cl inter­actions [2.793 (5) Å] result in the formation of a chain along the *a* axis.

## Related literature

For background to the use of unsymmetrical tridentate Schiff base ligands and their hydrogenated derivatives in coordin­ation chemistry for the assembly of alkoxo-or phenoxo-bridged clusters and polymers, see: Koizumi *et al.* (2005 ▶); Boskovic *et al.* (2003 ▶); Oshiob *et al.* (2005 ▶). For related structures, see: Bluhm *et al.* (2003 ▶); Kannappan *et al.* (2005 ▶); Sun *et al.* (2005 ▶).
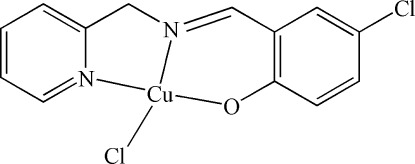


## Experimental

### 

#### Crystal data

[Cu(C_13_H_10_ClN_2_O)Cl]
*M* *_r_* = 344.67
Orthorhombic, 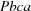

*a* = 7.7975 (11) Å
*b* = 13.638 (2) Å
*c* = 24.854 (4) Å
*V* = 2643.1 (7) Å^3^
*Z* = 8
Mo *K*α radiation
μ = 2.05 mm^−1^
*T* = 293 K
0.15 × 0.12 × 0.09 mm

#### Data collection

Bruker APEXII diffractometer
Absorption correction: multi-scan (*SADABS*; Sheldrick, 2008*a* ▶) *T*_min_ = 0.749, *T*_max_ = 0.837
12098 measured reflections
2325 independent reflections
1580 reflections with *I* > 2σ(*I*)
*R*_int_ = 0.053

#### Refinement

*R*[*F* ^2^ > 2σ(*F* ^2^)] = 0.038
*wR*(*F* ^2^) = 0.098
*S* = 1.02
2325 reflections
172 parameters
H-atom parameters constrained
Δρ_max_ = 0.29 e Å^−3^
Δρ_min_ = −0.33 e Å^−3^

### 

Data collection: *APEX2* (Bruker, 2004 ▶); cell refinement: *SAINT-Plus* (Bruker, 2001 ▶); data reduction: *SAINT-Plus*; program(s) used to solve structure: *SHELXS97* (Sheldrick, 2008*b* ▶); program(s) used to refine structure: *SHELXL97* (Sheldrick, 2008*b* ▶); molecular graphics: *SHELXTL* (Sheldrick, 2008*b* ▶); software used to prepare material for publication: *SHELXTL*.

## Supplementary Material

Crystal structure: contains datablock(s) I, global. DOI: 10.1107/S1600536812013359/hg5195sup1.cif

Structure factors: contains datablock(s) I. DOI: 10.1107/S1600536812013359/hg5195Isup2.hkl

Additional supplementary materials:  crystallographic information; 3D view; checkCIF report

## Figures and Tables

**Fig. 1.**
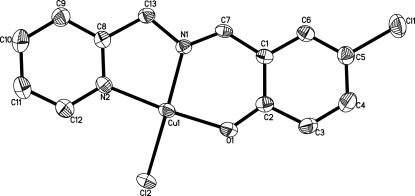
View of the title compound with the atom-labelling scheme Displacement ellipsoids are drawn at the 30% probability level. All H-atoms are omitted for clarity.

## supplementary crystallographic information

### Comment

Schiff base complexes have all along attracted much attention due to their interesting structures and wide potential applications. Recently, the relative flexible unsymmetrical tridentate Schiff base ligands and their hydrogenerated derivatives have been introduced into the coordination chemistry to assemble alkoxo-or phenoxo-bridged clusters and polymers with beautiful molecular structures and interesting magnetic properties (Koizumi *et al.*, 2005; Boskovic *et al.*, 2003; Oshiob *et al.*, 2005). Herein, we report the structure of a new copper complex based on an unsymmetric tridentate Schiff base ligand.

The molecular structure of title compound is shown in Fig. 1. The Cu ion is four coordinate forming a slightly distorted square planar coordination sphere, in which three positions are occupied by two N atoms and one O atom from the asymmetric tridentate Schiff base ligand, and the other one coming from a coordinated chloride ion. The CuN_2_O unit is located in a well plane with the mean deviation of 0.0035 (3) Å, while the chloro ion is obvious out of the above plane with deviation value 0.1249 (5) Å. The bond distances of Cu—O, Cu—N and Cu—Cl are in the normal range compared to the reported complexes containing the analogous unsymmetrical tridentate Schiff base ligands (Bluhm *et al.*, 2003; Kannappan, *et al.*, 2005; Sun *et al.*, 2005). It is worth noting that the asymmetric unit can be linked into one dimensional double chain structure by the weak Cu···Cl intermolecular interactions.

### Experimental

The Schiff base was obtained by condensation 2-(aminomethyl)pyridine and 5-chloro-2-hydroxy-benzaldehyde with the ratio 1:1 in methanol. The synthesis of the title complex was carried out by the reaction of CuCl_2_.6H_2_O and the Schiff-base ligand (1:1, molar ratio) in methanol under the stirring condition at room temperature. The filtrated solution was allowed to partial evaporation and blue single crystals suitable for X-ray diffraction were afforded with the yield about 60% sevral days later.

### Refinement

All the H atoms bonded to the C atoms were placed using the HFIX commands in *SHELXL-97*, with C—H distances of 0.93 and 0.96 Å, and were allowed for as riding atoms with *U*_iso_(H) = 1.2*U*_eq_(C).

### Crystal data

**Table:**

[Cu(C_13_H_10_ClN_2_O)Cl]	*F*(000) = 1384
*M**_r_* = 344.67	*D*_x_ = 1.732 Mg m^−^^3^
Orthorhombic, *P**b**c**a*	Mo *K*α radiation, λ = 0.71073 Å
Hall symbol: -P 2ac 2ab	Cell parameters from 1346 reflections
*a* = 7.7975 (11) Å	θ = 2.8–26.3°
*b* = 13.638 (2) Å	µ = 2.05 mm^−^^1^
*c* = 24.854 (4) Å	*T* = 293 K
*V* = 2643.1 (7) Å^3^	Block, blue
*Z* = 8	0.15 × 0.12 × 0.09 mm

### Data collection

**Table:**

Bruker APEXII diffractometer	2325 independent reflections
Radiation source: fine-focus sealed tube	1580 reflections with *I* > 2σ(*I*)
Graphite monochromator	*R*_int_ = 0.053
φ and ω scans	θ_max_ = 25.0°, θ_min_ = 1.6°
Absorption correction: multi-scan (*SADABS*; Sheldrick, 2008*a*)	*h* = −9→9
*T*_min_ = 0.749, *T*_max_ = 0.837	*k* = −14→16
12098 measured reflections	*l* = −27→29

### Refinement

**Table:**

Refinement on *F*^2^	Primary atom site location: structure-invariant direct methods
Least-squares matrix: full	Secondary atom site location: difference Fourier map
*R*[*F*^2^ > 2σ(*F*^2^)] = 0.038	Hydrogen site location: inferred from neighbouring sites
*wR*(*F*^2^) = 0.098	H-atom parameters constrained
*S* = 1.02	*w* = 1/[σ^2^(*F*_o_^2^) + (0.0418*P*)^2^ + 1.4463*P*] where *P* = (*F*_o_^2^ + 2*F*_c_^2^)/3
2325 reflections	(Δ/σ)_max_ = 0.006
172 parameters	Δρ_max_ = 0.29 e Å^−^^3^
0 restraints	Δρ_min_ = −0.33 e Å^−^^3^

### Special details

**Table:**

Geometry. All e.s.d.'s (except the e.s.d. in the dihedral angle between two l.s. planes) are estimated using the full covariance matrix. The cell e.s.d.'s are taken into account individually in the estimation of e.s.d.'s in distances, angles and torsion angles; correlations between e.s.d.'s in cell parameters are only used when they are defined by crystal symmetry. An approximate (isotropic) treatment of cell e.s.d.'s is used for estimating e.s.d.'s involving l.s. planes.
Refinement. Refinement of *F*^2^ against ALL reflections. The weighted *R*-factor *wR* and goodness of fit *S* are based on *F*^2^, conventional *R*-factors *R* are based on *F*, with *F* set to zero for negative *F*^2^. The threshold expression of *F*^2^ > σ(*F*^2^) is used only for calculating *R*-factors(gt) *etc*. and is not relevant to the choice of reflections for refinement. *R*-factors based on *F*^2^ are statistically about twice as large as those based on *F*, and *R*- factors based on ALL data will be even larger.

### Fractional atomic coordinates and isotropic or equivalent isotropic displacement parameters (Å^2^)

**Table:**

	*x*	*y*	*z*	*U*_iso_*/*U*_eq_	
Cu1	0.10863 (6)	0.97468 (3)	0.248134 (18)	0.03844 (17)	
Cl1	0.43484 (19)	0.64978 (10)	0.45552 (5)	0.0755 (4)	
Cl2	−0.09768 (12)	1.09117 (7)	0.25915 (4)	0.0441 (3)	
O1	0.1221 (4)	0.9585 (2)	0.32421 (11)	0.0504 (8)	
N1	0.2305 (4)	0.8517 (2)	0.23433 (11)	0.0349 (7)	
N2	0.1071 (4)	0.9820 (2)	0.16720 (13)	0.0407 (8)	
C1	0.2746 (5)	0.8043 (3)	0.32695 (15)	0.0358 (9)	
C2	0.1939 (5)	0.8871 (3)	0.35062 (16)	0.0399 (10)	
C3	0.1894 (6)	0.8900 (3)	0.40777 (17)	0.0524 (12)	
H3	0.1355	0.9426	0.4246	0.063*	
C4	0.2610 (6)	0.8186 (3)	0.43873 (17)	0.0564 (12)	
H4	0.2573	0.8231	0.4760	0.068*	
C5	0.3394 (5)	0.7391 (3)	0.41436 (17)	0.0472 (11)	
C6	0.3446 (5)	0.7312 (3)	0.36013 (16)	0.0414 (10)	
H6	0.3953	0.6764	0.3446	0.050*	
C7	0.2900 (5)	0.7931 (3)	0.27008 (15)	0.0343 (9)	
H7	0.3488	0.7381	0.2578	0.041*	
C8	0.1920 (5)	0.9084 (3)	0.14267 (15)	0.0392 (10)	
C9	0.2162 (6)	0.9091 (3)	0.08752 (17)	0.0544 (12)	
H9	0.2736	0.8576	0.0709	0.065*	
C10	0.1555 (6)	0.9855 (4)	0.05748 (19)	0.0655 (14)	
H10	0.1727	0.9868	0.0205	0.079*	
C11	0.0687 (6)	1.0606 (4)	0.08264 (19)	0.0646 (13)	
H11	0.0268	1.1134	0.0630	0.077*	
C12	0.0453 (6)	1.0559 (3)	0.13728 (17)	0.0535 (12)	
H12	−0.0157	1.1057	0.1542	0.064*	
C13	0.2559 (5)	0.8271 (3)	0.17772 (14)	0.0407 (10)	
H13A	0.1948	0.7671	0.1693	0.049*	
H13B	0.3770	0.8163	0.1709	0.049*	

### Atomic displacement parameters (Å^2^)

**Table:**

	*U*^11^	*U*^22^	*U*^33^	*U*^12^	*U*^13^	*U*^23^
Cu1	0.0423 (3)	0.0278 (3)	0.0452 (3)	0.0030 (2)	0.0022 (3)	−0.0004 (2)
Cl1	0.1064 (11)	0.0612 (8)	0.0589 (7)	0.0109 (8)	−0.0199 (7)	0.0129 (6)
Cl2	0.0358 (5)	0.0300 (5)	0.0665 (7)	0.0016 (4)	0.0006 (5)	−0.0014 (5)
O1	0.065 (2)	0.0367 (17)	0.0496 (17)	0.0140 (15)	0.0072 (15)	0.0007 (14)
N1	0.0397 (19)	0.0258 (17)	0.0392 (18)	−0.0030 (15)	0.0042 (15)	−0.0022 (14)
N2	0.040 (2)	0.0336 (19)	0.0484 (19)	−0.0033 (17)	−0.0025 (16)	0.0041 (16)
C1	0.034 (2)	0.028 (2)	0.045 (2)	−0.0045 (17)	0.0006 (18)	−0.0028 (18)
C2	0.041 (2)	0.032 (2)	0.047 (2)	−0.0053 (19)	0.004 (2)	−0.0024 (19)
C3	0.065 (3)	0.044 (3)	0.048 (3)	0.007 (2)	0.006 (2)	−0.007 (2)
C4	0.070 (3)	0.058 (3)	0.042 (2)	−0.005 (3)	0.002 (2)	−0.003 (2)
C5	0.052 (3)	0.041 (3)	0.048 (3)	−0.003 (2)	−0.004 (2)	0.003 (2)
C6	0.044 (3)	0.030 (2)	0.050 (3)	−0.0025 (18)	−0.0013 (19)	−0.003 (2)
C7	0.031 (2)	0.024 (2)	0.048 (2)	−0.0001 (17)	0.0022 (18)	−0.0048 (18)
C8	0.038 (2)	0.035 (2)	0.044 (2)	−0.0071 (19)	−0.0029 (19)	0.000 (2)
C9	0.063 (3)	0.051 (3)	0.049 (3)	0.000 (2)	0.000 (2)	−0.002 (2)
C10	0.078 (4)	0.074 (4)	0.045 (3)	−0.004 (3)	−0.005 (2)	0.009 (3)
C11	0.074 (4)	0.060 (3)	0.059 (3)	0.002 (3)	−0.008 (3)	0.016 (3)
C12	0.057 (3)	0.045 (3)	0.058 (3)	0.001 (2)	−0.001 (2)	0.004 (2)
C13	0.044 (3)	0.036 (2)	0.042 (2)	0.0012 (19)	0.001 (2)	−0.0045 (18)

### Geometric parameters (Å, º)

**Table:**

Cu1—O1	1.907 (3)	C4—C5	1.384 (6)
Cu1—N1	1.958 (3)	C4—H4	0.9300
Cu1—N2	2.014 (3)	C5—C6	1.353 (5)
Cu1—Cl2	2.2775 (11)	C6—H6	0.9300
Cl1—C5	1.756 (4)	C7—H7	0.9300
O1—C2	1.300 (4)	C8—C9	1.383 (5)
N1—C7	1.282 (4)	C8—C13	1.495 (5)
N1—C13	1.460 (4)	C9—C10	1.367 (6)
N2—C12	1.342 (5)	C9—H9	0.9300
N2—C8	1.348 (5)	C10—C11	1.377 (6)
C1—C6	1.404 (5)	C10—H10	0.9300
C1—C2	1.421 (5)	C11—C12	1.372 (6)
C1—C7	1.427 (5)	C11—H11	0.9300
C2—C3	1.422 (5)	C12—H12	0.9300
C3—C4	1.362 (6)	C13—H13A	0.9700
C3—H3	0.9300	C13—H13B	0.9700
			
O1—Cu1—N1	92.74 (12)	C5—C6—C1	121.1 (4)
O1—Cu1—N2	175.25 (13)	C5—C6—H6	119.4
N1—Cu1—N2	82.55 (13)	C1—C6—H6	119.4
O1—Cu1—Cl2	90.03 (9)	N1—C7—C1	126.1 (4)
N1—Cu1—Cl2	164.03 (10)	N1—C7—H7	117.0
N2—Cu1—Cl2	94.67 (10)	C1—C7—H7	117.0
C2—O1—Cu1	127.7 (3)	N2—C8—C9	120.6 (4)
C7—N1—C13	118.4 (3)	N2—C8—C13	116.9 (3)
C7—N1—Cu1	126.0 (3)	C9—C8—C13	122.5 (4)
C13—N1—Cu1	115.6 (2)	C10—C9—C8	120.0 (4)
C12—N2—C8	119.0 (4)	C10—C9—H9	120.0
C12—N2—Cu1	126.4 (3)	C8—C9—H9	120.0
C8—N2—Cu1	114.3 (3)	C9—C10—C11	119.3 (5)
C6—C1—C2	119.6 (4)	C9—C10—H10	120.4
C6—C1—C7	118.2 (4)	C11—C10—H10	120.4
C2—C1—C7	122.2 (3)	C12—C11—C10	118.7 (5)
O1—C2—C1	125.2 (4)	C12—C11—H11	120.7
O1—C2—C3	118.2 (4)	C10—C11—H11	120.7
C1—C2—C3	116.5 (4)	N2—C12—C11	122.4 (4)
C4—C3—C2	122.3 (4)	N2—C12—H12	118.8
C4—C3—H3	118.8	C11—C12—H12	118.8
C2—C3—H3	118.8	N1—C13—C8	110.2 (3)
C3—C4—C5	119.6 (4)	N1—C13—H13A	109.6
C3—C4—H4	120.2	C8—C13—H13A	109.6
C5—C4—H4	120.2	N1—C13—H13B	109.6
C6—C5—C4	120.8 (4)	C8—C13—H13B	109.6
C6—C5—Cl1	120.8 (3)	H13A—C13—H13B	108.1
C4—C5—Cl1	118.4 (3)		
